# Exploring DC Power Quality Measurement and Characterization Techniques

**DOI:** 10.3390/s25196043

**Published:** 2025-10-01

**Authors:** Yara Daaboul, Daniela Istrate, Yann Le Bihan, Ludovic Bertin, Xavier Yang

**Affiliations:** 1 Laboratoire National de Métrologie et D’essais (LNE), 78190 Trappes, France; daniela.istrate@lne.fr; 2 GeePs, Laboratoire de Génie Electrique Et Electronique de Paris, CNRS, CentraleSupélec, University Paris-Saclay, Sorbonne University, 91190 Gif-sur-Yvette, France; yann.le-bihan@centralesupelec.fr; 3 EDF—R&D, Electricité de France, 91190 Saclay, France; ludovic-g.bertin@edf.fr (L.B.); xavier.yang@edf.fr (X.Y.)

**Keywords:** LVDC grids, DC distortions, DC ripple, measurement chain, accuracy

## Abstract

Within the modernizing energy infrastructure of today, the integration of renewable energy sources and direct current (DC)-powered technologies calls for the re-examination of traditional alternative current (AC) networks. Low-voltage DC (LVDC) grids offer an attractive way forward in reducing conversion losses and simplifying local power management. However, ensuring reliable operation depends on a thorough understanding of DC distortions—phenomena generated by power converters, source instability, and varying loads. Two complementary traceable measurement chains are presented in this article with the purpose of measuring the steady-state DC component and the amplitude and frequency of the distortions around the DC bus with low uncertainties. One chain is optimized for laboratory environments, with high effectiveness in a controlled setup, and the other one is designed as a flexible and easily transportable solution, ensuring efficient and accurate assessments of DC distortions for field applications. In addition to our hardware solutions fully characterized by the uncertainty budget, we present the measurement method used for assessing DC distortions after evaluating the limitations of conventional AC techniques. Both arrangements are set to measure voltages of up to 1000 V, currents of up to 30 A, and frequency components of up to 150–500 kHz, with an uncertainty varying from 0.01% to less than 1%. This level of accuracy in the measurements will allow us to draw reliable conclusions regarding the dynamic behavior of future LVDC grids.

## 1. Introduction

Over the past two decades, the paradigm shift in energy generation and consumption has brought the spotlight to local DC grids as an expansion of traditional AC grids. As native DC technologies such as renewable energy sources, LED lighting, storage units, and electric vehicles have gained greater usage, the decrease in energy efficiency caused by AC–DC conversions have also gained attention. Therefore, grid operators have come to view LVDC grids (up to 1500 V_DC_ [1]) as a mean of reducing conversion losses. However, more investigations are needed to find their potential benefits, including lower distribution losses, improved voltage stability, fewer substations, and simpler integration of renewable energy sources [2,3,4,5]. Nevertheless, to unleash the full potential of DC grids, several key research areas need to be further explored. Among these challenges, we highlight the following: the still-limited understanding of DC grid dynamics, the need for in-depth studies on appropriate protection strategies, the long-term aging of components in a DC environment, the absence of clear standards for electromagnetic compatibility levels, and the accurate measurement of power and energy for billing purposes. Indeed, most electricity meters are designed based on AC standards, which gives perspective to the need for an exhaustive analysis of metrological challenges that are particular to DC grids. Mastering these aspects and developing the necessary expertise fundamentally rely on the ability to perform reliable measurements, as they provide the foundation for understanding LVDC network dynamics. Additionally, when mentionning reliable measurements, two critical dimensions emerge: the performance of the measurement chain, and the measurement and data processing methods, which ensure that the results obtained accurately reflect the reality.

To delve into these measurement challenges, we conducted the study presented in this paper in five different sections: Section 2 presents an overview of the state of the art and recent advancements in LVDC research, together with our own examination of data obtained from previous measurement campaigns, laying a solid foundation for our study. Section 3 outlines the two measurement setups proposed, designed to meet specific criteria, and presents the measurement method used based on the critical evaluation of the standardized method within the AC domain. Section 4 focuses on the various sources of uncertainty for each measurand, with an evaluation of the uncertainty budget. Finally, Section 5 and Section 6 provide the conclusions and discussions, highlighting problems still to be solved and potential future research paths.

## 2. Research Overview and Preliminary Analysis

### 2.1. Advancements in LVDC Research

It is important to highlight that there have been advancements in research on LVDC networks over the past few years, including pilot projects, standardization efforts, measurement campaigns, and attempts in the context of a European project to propose DC measurement systems and setups for DC power quality studies, all aimed at enhancing the understanding and evaluation of LVDC grids.

#### 2.1.1. Pilot Projects for LVDCs

Numerous solutions and pilot projects have been initiated to explore the feasibility and benefits of low-voltage DC (LVDC) networks: LV-Engine project in the United Kingdom [6], DC distribution projects in South Korea [7], field test environments for LVDC distribution in Finland [8], Suzhou Tongli LVDC demonstrator in China [9]. These pilot projects assessed the practical implementation of DC grids in residential, industrial, and commercial settings. They also demonstrated how DC distribution grids can enhance the efficiency, stability, and renewable energy integration in the grid, reduce transformation expenses, and allow hybrid AC/DC systems. However, additional research, standardization, and pilot schemes are needed to address operational safety, long-term impacts, and commercialization. 

#### 2.1.2. Standardization Efforts

Standardization efforts for DC power quality in LVDC grids are at an early stage but are progressing through academic initiatives, projects, and research publications. Barros et al. (2022) [10] identified voltage ripple, DC distortions, and voltage dips and swells as the most critical PQ disturbances in LVDC networks. Khilnani et al. (2020) [11], Mariscotti et al. (2021) [12], and Van den Brom et al. (2023) [13] reviewed existing AC PQ standards and argued that they could be directly extended to DC grids, in particular suggesting the use of the AC harmonic standard IEC 61000-4-7: 2002 [14] for their equivalent DC distortions in the 0–2 kHz range and the IEC 61000-4-30: 2015 [15] for supraharmonics in the 2–150 kHz range. In contrast, Yang et al. (2022) [16] proposed a different approach based on an alternative spectral decomposition, to better capture and classify DCPQ disturbances; they recommend two frequency ranges, the 0–9 kHz range referred to low frequency disturbances quantified with the IEC 61000-4-7:2002 [14] standard, and the >9 kHz range referred to conducted disturbances quantified with the CISPR-16 [17] standard initially developed for radio-communication purposes. Additionally, Duan et al. (2024) [18] proposed a novel method for classifying DCPQ disturbances by integrating advanced feature extraction with an optimized neural network.

At the international level, the IEC has created the System Committee on LVDC (SyC LVDC) and released the IEC TR 63282:2024 [19], which not only defines nominal LVDC voltage levels but also proposes preliminary requirements for DCPQ. The technical report defines DC distortions as: “multifrequency AC components, also referred to as DC distortions, within DC voltage and current, that are mainly originated by the switching or regulation of semi-conductors converters, by the connection of non-linear loads and their variations within the utilization equipment, and by the rectification of an AC voltage signal at the DC grid entry AC/DC converter”. The TR 63282 recommends the adoption of the spectral decomposition proposed in [16], and characterizes the DC distortions by the following:

The distortion spectrum: it quantifies the DC component and the amplitude and phase of each frequency present in the DC voltage or current. Spectral analysis of DC distortion is performed during each measurement duration of T_w_ through a Discrete Fourier Transform (DFT) calculation. So far, based on IEC proposals, T_w_ is set to 200 ms [19].

RMS ripple (in %), given by (1) and (2): it is the RMS value of all voltage variations divided by the DC voltage calculated for the duration T_w_, with U_rms_ and U_DC_ calculated from the distortion spectrum:

(1) $$Ripple_{RMS}\;\left(\%\right)=\;\frac{\sqrt{U_{rms}^{2}-U_{DC}^{2}}}{U_{DC}}\;\cdot 100$$

(2) $$U_{rms}=\sqrt{\sum_{i=0}^{m}U_{i}^{2}}$$

where m represents the maximum index in the 0–9 kHz range within the DFT window, U_i_ is the RMS value of the frequency component included in the 0–9 kHz spectrum such as U_0_ = U_DC_, and U_1_ is the RMS value of the frequency component at 5 Hz, since a 200 ms window results in a frequency step of 5 Hz in the spectrum.

In our study, we adopted the IEC TR 63282 recommendations for DC power quality, as they currently represent the closest initiative to an established standard for DC distortions. We focus on analyzing distortions across a wide frequency range, up to 150–500 kHz. Accordingly, m is defined as the maximum index within the studied frequency range.

#### 2.1.3. Measurement Systems for DCPQ in LVDC Networks

The limited understanding of power quality in LVDC networks, and its implications for accurate DC electricity metering, motivated the launch of the joint European metrology research project “20NRM03 DC Grids” [20]. This initiative seeked to establish traceable measurement systems for DCPQ parameters, providing a foundation for standardization of future DC grids. The measurement systems developed in this project are summarized in Table 1, which reports the uncertainties for distortion measurements in current and voltage. Table 1 shows three measurement systems developed by the following: VSL the National Metrology Institute in the Netherlands, PTB the National Metrology Institude in Germany, and METAS the Federal Institute of Metrology in Switzerland. Each measurement system aimed at measuring generated DC voltage and current with AC components superposed on the DC component to represent DC distortions.

In our work, as will be detailed in the following paragraphs, we have attempted to propose two complementary measurement setups for quantifying DC distortions up to 150–500 kHz, including a detailed uncertainty budget, and we aimed to position our contribution with respect to these advancements and proposals.

#### 2.1.4. Measurement Campaigns

Several measurement campaigns have been conducted to assess power quality disturbances, including voltage and current distortions, voltage fluctuations, inrush currents, and harmonic content, in VSL [13], CIRCE, the Research Center for Energy Consumption in Spain [21], and EDF R&D in China [22]. Findings from these campaigns contribute to refining measurement methodologies, improving sensor designs, and establishing more accurate DC metrology frameworks. On the basis of these enhancements, we made a decision to further investigate data obtained from some campaigns in an attempt to define the metrology limits and challenges that could influence DC phenomena characterization.

### 2.2. Analysis of Data from Measurement Campaigns

**Figure 1:**
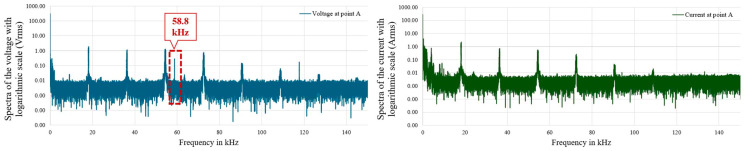
Voltage and current spectra from the Chinese measurement campaign.

As previously stated, to gain deeper insights into the measurement challenges associated with identifying and characterizing DC distortions, we analyzed the measurement campaign data supplied by EDF R&D China. During this analysis, we encountered cases highlighting the influence of the measurement setup on the results. For example, we found that the components of the measurement chain can, in certain cases, interfere with the signals being measured and disturb the measurement results. For instance, electromagnetic radiation from power supplies, cables, and chosen probes can be mistakenly identified as actual disturbances in the LVDC network by appearing as significant frequency components in the distortion spectrum. Indeed, as shown in Figure 1, in the spectrum obtained for measured voltage on the DC bus, a frequency component appears at 58.8 kHz for all the grid configurations. It was concluded that the 58.8 kHz disturbance originates from the voltage probe or the measurement system’s power supply. This conclusion is supported by two observations: first, the disturbance was consistently present in the measured voltage spectra across all tested grid configurations—including varying the operating point of the photovoltaic (PV) source—yet it never appeared in the corresponding current spectra. Second, when comparing the measurements with the spectra obtained from modeling the demonstrator in the power system simulator PSCAD, this component was absent, further indicating that it does not originate from the actual grid but from the measurement setup.

This is crucial because, in this new domain of DC networks, the frequency components present in the spectrum cannot be anticipated, making it possible to mistakenly attribute any significant component to a grid-related phenomenon.

Second, an experiment conducted in our labs using a DC source, whose distortion frequencies and levels are well known, revealed the impact of noise from the acquisition system. At times, the low-amplitude disturbances of the source were masked by the electronic noise of the measuring instrument, particularly when the range of the source was set at high levels, requiring the use of high measurement ranges. Since the definition of what constitutes a “significant level of disturbance” in DC domains is still quite uncertain due to the absence of clear standards for electromagnetic compatibility emission levels, it is crucial to choose a measurement chain with low noise levels so it can be capable of identifying different amplitudes of distortions.

In summary, the work previously carried out on LVDC grids in terms of standardization, demonstration sites, measurement systems, and measurement campaigns has provided valuable insights into the complexities of accurately measuring DC distortions. Analysis of data from measurement campaigns also reinforced the necessity for a thoroughly understood and controlled measurement chain and method. These examples show that without proper knowledge and control of the measurement setup, we can obtain either incomplete or biased results. Hence, the main challenge nowadays is to properly identify and measure DC distortions with small uncertainty, and this can be achieved through the development of measurement chains and techniques, as outlined in Section 3.

## 3. Measurement Systems

The necessity for developing a dedicated measurement chain in the DC domain arises from the various challenges and limitations it faces nowadays:

‑ First, measuring both the high-magnitude DC component (up to 1000 V) and the much smaller AC distortion parameters (ranging from hundreds of millivolts to tens of volts) with the same accuracy and setup presents a significant challenge, as the distortion amplitude can be up to 1000 times smaller than the DC component.
‑ Second, acquisition systems inherently require a trade-off between resolution and bandwidth, and the choice of sensors introduces another compromise between linearity and bandwidth, meaning that achieving high accuracy for DC component measurements often comes at the expense of frequency response, limiting the chain’s ability to simultaneously capture fine details of the AC distortions while maintaining accurate DC measurements.
‑ Finally, the literature on LVDC measurement systems remains limited, with even fewer works addressing the traceable characterization of such systems. Only a limited number of measurement setups have been reported, as detailed earlier.

Therefore, considering these limitations and the challenges we identified through the analysis of the campaigns data in Section 2, our objective is to propose a setup capable of accurately capturing the true behavior of the DC phenomena by building on the existing measurement setups and improving specific parameters. Thus, recognizing the need to address both high-accuracy laboratory measurements and flexible field assessments, we developed two distinct measurement chains, each optimized for a different environment and based on certain technical criteria: The first setup, designed for laboratory use, prioritizes low uncertainty by leveraging accurate calibration and controlled conditions, though it sacrifices some flexibility. On the other hand, the second setup uses flexible and robust components to withstand the uncontrolled character of the on-site networks, but presents higher uncertainties. This dual approach reflects our broader objective of accurately assessing on-site distortions in addition to laboratory measurements, with specific criteria:

- The incorporation of wideband transducers to accurately capture emissions extending into the hundreds of kilohertz. Indeed, this high frequency band is gaining increasing interest with the rising integration of power electronics in electrical grids.
- The use of low-noise acquisition systems to ensure precise detection of low-amplitude distortions.
- The improvement of spectral and temporal resolution in the acquisition process, achieved through higher sampling rates and acquisition systems resolution.

### 3.1. Laboratory Measurement System

**Figure 2:**
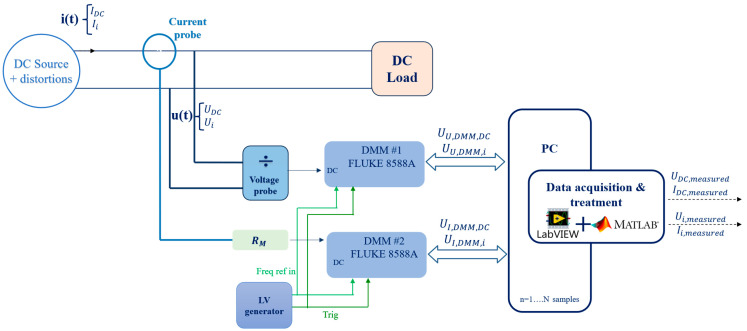
Laboratory measurement system for DC distortions.

The schematic illustrated in Figure 2 shows a DC source supplying a DC load. The voltage provided by the source and the current flowing through the load contain DC distortions. The acquisition system is based on two digital multimeters (DMMs) of type Fluke 8588A [23]. Both DMMs offer 18 bits resolution, with multiple ranges (100 mV, 1 V, 10 V, 100 V, and 1000 V) and a sampling frequency as high as 5 MHz. They are both synchronized by applying a 10 MHz clock reference and using a 100 Hz signal as the external trigger. The measured signals are sampled at a 1 MHz rate and acquired using a LabVIEW program, while signal processing is performed through a MATLAB-developed interface.

For the voltage probe in Figure 2, the voltage signal is attenuated through a voltage divider that accepts a maximum input voltage of 1000 V and gives a maximum output of 0.8 V, allowing us to benefit from the most accurate ranges of the DMM.

To measure current, a current probe of type LEM IN 100-S [24] is utilized; its output current is converted to an equivalent proportional voltage via a resistor RM and is then read by the DMM. The probe characteristics are listed: 100 A peak current; 500:1 division ratio, and DC—2 MHz bandwidth.

As the acquisition systems (DMMs) are designed to measure voltages, their outputs from voltage or current measurements are expressed in volts (for example, U_I,DMM,DC_ is the DC voltage output of the measured current via the DMM). In the interface, the processed signals are then scaled using the division and conversion ratios determined during calibration. The outputs of the setup are the value of the DC component (U_DC,measured_ for the measured voltage and I_DC,measured_ for the current), as well as the amplitude of the various distortions measured around the DC bus (U_i,measured_, I_i,measured_). The entire measurement setup was characterized and metrologically calibrated as described in Section 4.

### 3.2. On-Site Measurement System

**Figure 3:**
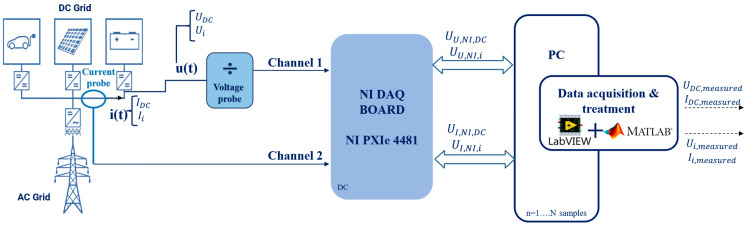
On-site measurement system for DC distortions.

Several constraints concerning flexibility, safety of equipment, and portability have to be considered when designing a measurement system for field applications. The on-site measurement system is illustrated in Figure 3 and includes the use of voltage, and current sensors to adapt the signal according to the inputs of the acquisition system: a NI PXIe 4481 card. The choice of this acquisition system relies on its advantages with respect to the laboratory system: the six acquisition channels available on the NI PXI card, its more compact shape making it more easily portable, and its better resolution (24 bits) [25].

The limited input range (±10 V) of the NI card imposes the use of a voltage probe. In contrast with the laboratory environment where a resistive and high-precision voltage divider might be used to reach low measurement uncertainties, in uncontrolled field environments, voltage surges can exceed the acceptable limits of the divider, leading to excessive power dissipation and potential damage. The voltage probes, as used in our on-site system, are designed to tolerate temporary overvoltage by gradually saturating, providing better protection. For the current measurements, the current probe used in the laboratory setup is highly precise and requires a resistor to convert the current being measured into a voltage suitable for the acquisition system. This adds complexity to the setup. In addition, due to its circular design, the LEM probe requires one to unplug cables in order to insert them into the probe which makes it less convenient for on-site tests. Thus, openable current clamps are chosen in this case.

The details of the components that constitute the on-site system are the following:

The NI PXIe 4481 employs a delta-sigma analog-to-digital converter (ADC) of 24-bit resolution. This type of converter transforms analog signals to digital samples using sigma-delta modulation techniques, which allow high accuracy and low noise levels. The board can handle a sampling rate of 1 MHz and accommodates both differential and pseudo-differential input configurations. One of the inputs in pseudo-differential mode is connected to a reference voltage (in this case, chassis ground) with a 50 ohm input impedance between the negative input and ground, which helps to reduce common-mode noise. Another key advantage of this acquisition system is its flexibility. It is a multi-board, multi-channel system that enables real-time acquisitions, an essential feature for our quality studies, by achieving a bufferised acquisition-to-display delay of less than 200 ms. Additionally, it is fully controlled by a LabVIEW program, offering significant flexibility in terms of configurations and synchronization types.

The voltage signal is attenuated through a voltage probe. We thoroughly tested four different types and retained the best two in terms of performance, a MTX-1032 [26] that can measure up to a maximum of 400 V with a division ration of 100:1 and a bandwidth of DC—30 MHz; and a Hioki 9322 [27] that can measure up to 2000 V with a division ration of 1000:1 and a bandwidth of DC—10 MHz. We mention two possible probes for the on-site system because the MTX, uncapable of measuring up to our target level of 1000 V, is the preferred choice according to the following criteria:

- While evaluating the noise level of the probes combined with the NI board, we found a higher noise level of 68 dBµV for the Hioki compared to 45 dBµV for the MTX.
- The MTX probe is evaluated in multiple field campaigns, such as testing protection schemes for DC systems with DC/DC and AC/DC converters, characterization of EV dynamics under various disturbance conditions, etc. Thus, its robustness and stability established under power electronics disturbances are extensively validated. Therefore, the MTX is chosen for the uncertainty calculations conducted in Section 5.

However, to measure voltages above 400 V, we can use the Hioki probe.

To measure current, a Hioki CT6710 current clamp [28] is utilized; its characteristics are as follows: 30 A peak current; 0.1 V/A conversion ratio; and DC-50 MHz bandwidth.

Similarly to the laboratory setup, the NI PXIe is designed to measure voltages, its outputs from voltage or current measurements are expressed in volts (for example, U_I,NI,DC_ is the DC voltage output of the measured current via the NI board). The outputs of the setup are the DC component (U_DC,measured_ for the measured voltage and I_DC,measured_ for the current), as well as the amplitude of the various distortions (U_i,measured_, I_i,measured_).

### 3.3. Measurement Method

In order to develop techniques for DC distortion measurements, one has to find out whether methods established and normalized for AC can be applied for DC. For AC, the standard approaches utilize a DFT with non-overlapping sliding rectangular windows for an observation time of 10 or 12 cycles of the signal (equal to 200 ms), which is effective due to synchronization with the fundamental AC frequency (50 or 60 Hz). Indeed, in the case of a 50 Hz fundamental frequency, because the measured signals are synchronized with this 50 Hz, their truncation over 200 ms contains an integer number of periods, and therefore DFT gives exact results. However, in DC, the frequencies of the measured phenomena are neither controlled nor synchronous with 50 Hz, and therefore the samples acquired do not include an integer number of periods of the signal. In this case, truncation (or in other words, the use of a rectangular window) introduces artifacts or spectral leakage, as explained in [29,30]. Thus, the spectrum obtained is not the actual spectrum of the original signal. Therefore, in DC, we conclude that the use of an observation window different from the rectangular one is required in order to reduce the effects of spectral leakage, since these windows have an amplitude that gradually decreases to zero at the ends and thus attenuates the discontinuities at the edges. Therefore, the most commonly used windows in signal processing applications, Hanning, Hamming, and Blackman are tested on a 350 V_DC_ signal with an AC component of 1673 Hz and 20 V which is not synchronized with the 200 ms observation window. For each windowed signal, amplitude accuracy, spectral leakage, and frequency resolution are quantified, and summarized in Table 2:

- Amplitude accuracy is quantified as the logarithmic ratio between the measured amplitude and the generated 20 V. Higher values reflect better amplitude accuracy.
- Spectral leakage is quantified as the logarithmic ratio between the maximum measured amplitude of the frequencies at ±25 Hz around the central frequency and the generated 20 V. Higher values reflect worse spectral leakage.
- Frequency resolution is quantified as the number of frequency components around the central frequency with amplitude losses lower than −3 dB relative to the 20 V. Higher results reflect worse frequency resolution.

**Table 2:** Window comparison for DCPQ.

	**Rectangular**	**Hanning**	**Hamming**	**Blackman**
Amplitude losses (dB)	−2.4	−2.7	−2.5	−3.5
Spectral leakage (dB)	−21.5	−44.8	−45.1	−63
Frequency resolution (number of frequency components)	1	4	4	5

Based on the results of Table 2, we noticed that the window with the best attenuation of the spectral leakage is the Blackman window and the one with the highest leakage is the rectangular, as previously mentioned. On the other hand, the Blackman shows the highest amplitude losses and lowest frequency resolution compared to the rectangular, which has the highest resolution and lowest amplitude losses. The Hamming and Hanning, compromising between the three criteria, have similar performances with slighty better leakage attenuation and amplitude accuracy for the Hamming. Therefore, it was chosen for our DFT calculations, and its amplitude errors are calculated, and incorporated in the total uncertainty budget, as shown in Section 4.1.2.

Thus, for DC, the proposed approach is to utilize a DFT with non-overlapping Hamming windows for an observation time of 200 ms to obtain the distortions spectrum.

## 4. Traceable Calibration and Uncertainty

To identify the quality and the correction factors of the proposed measurement setups, as well as to ensure their traceability, all the components were calibrated at LNE, the French National Metrology Laboratory. Reproducibility was tested at EDF’s research center for the current and voltage sensors. Input voltages and currents for system characterization were selected based on the literature as well as real grid measurement analysis. Moreover, all the measured quantities are traceable to the International System of Units by means of the calibration of the reference voltage source, standard current monitors, and time base, which are all linked to the French national primary standards.

In both measurement setups, the quantities of interest are the time-domain voltage and current waveforms of the LVDC grid, denoted as *u*(*t*) and *i*(*t*). These signals are acquired using the data acquisition system, and subsequently processed using a DFT. The DFT enables the decomposition of the signals into their frequency components where each component is characterized by an amplitude U_i_ or I_i_ with i from 0 to N/2—1 (corresponding to the positive frequency components of the N samples aquired). The DC component is given by U_0_ = U_DC_ and I_0_ = I_DC_, corresponding to the zero-frequency bin. The remaining components (i ≥1) represent the spectral content superposed on the DC bus and are quantified by their RMS amplitudes, U_i_ and I_i_, respectively. This frequency-domain decomposition of *u(t)* and *i(t),* represented in Figure 2 and Figure 3, allows for a detailed uncertainty analysis of each parameter, with its corresponding uncertainty contributions.

### 4.1. Laboratory Measurement System

The laboratory measurements involve temperature and humidity-controlled rooms with (23.0 ± 0.5) °C for the temperature regulation and (45 ± 5)% for the relative humidity.

#### 4.1.1. Uncertainty Calculation for U_DC_

The measured value of the DC voltage, U_DC,measured_, is associated with a measurement uncertainty that accounts for the combined relative uncertainties due to the acquisition system (DMM) given as $u_{R}(U_{U,DMM,DC})$, and due to the voltage probe given as $u_{R}(K_{VoltageProbe})$. By applying the law of propagation of uncertainties according to the guide to the expression of uncertainty, GUM [31], the combined relative uncertainty of U_DC,measured_ is obtained in (3):

(3) $${\;\;u}_{R}\left(U_{DC,measured}\right)=\;\sqrt{{\left(u_{R}\left(U_{U,DMM,DC}\right)\right)}^{2}+{\left(u_{R}\left(K_{VoltageProbe}\right)\right)}^{2}}$$

According to the GUM [31], Type A uncertainty is obtained through experimental measurements and statistical analysis, while Type B uncertainty is derived from non-statistical sources such as manufacturer specifications or prior knowledge. The uncertainty components considered for the budget are listed in Table 3.

The combined uncertainty related to U_U,DMM,DC_ and K_VoltageProbe_ are given in (4) and (5).

(4) $$u_{R}\left(U_{U,DMM,DC}\right)\;=\sqrt{u_{R}{\left(U_{CE,DMM}\right)}^{2}+u_{R}{\left(U_{D,DMM}\right)}^{2}+u_{R}{\left(U_{Q,DMM}\right)}^{2}+u_{R}{\left(U_{N,DMM}\right)}^{2}+u_{R}{\left(U_{T}\right)}^{2}+u_{R}{\left(U_{Z,DMM}\right)}^{2}+u_{R}{\left(U_{Offset,DMM}\right)}^{2}+u_{R}{\left(U_{Cables}\right)}^{2}}$$

(5) $$u_{R}\left(K_{VoltageProbe}\right)=\sqrt{u_{R}{\left(K_{CE}\right)}^{2}+u_{R}{\left(K_{D}\right)}^{2}+u_{R}{\left(K_{L}\right)}^{2}+u_{R}{\left(K_{Rep}\right)}^{2}}$$

The contributions to the global uncertainty budget are listed in Table 4. The expanded uncertainty is obtained, according to the GUM [31] from the combined standard uncertainty by applying a coverage factor k = 2 that corresponds to a coverage probability of 95.45% for a Gaussian distribution.

#### 4.1.2. Uncertainty Calculation for U_i_

The amplitudes of the distortions in u(t) are obtained in the frequency domain after applying the DFT, as described in Section 3. In this process, the combined uncertainty of U_i,measured_ includes an additional factor, *ε*_DFT_, which represents the standard uncertainty associated with amplitude attenuation due to the application of the Hamming window. This value is derived as the maximum relative attenuation in amplitude observed, due to the application of the window on different signals with different misalignments (non-synchronization) between the signals and the observation window, divided by $2\sqrt{3}$, in accordance with standard uncertainty evaluations for a rectangular distribution [31]. Although this type of error may initially appear systematic, it is not strictly systematic because the degree of amplitude attenuation depends not only on the type of window applied, which is known and fixed, but also on the spectral content of the signal, including the specific characteristics of the distortion and its degree of misalignment (non-synchronization) with the observation window. This misalignment is unknown, due to the uncertain and potentially non-periodic nature of DC signals.

Similarly to Equation (3), the combined uncertainty of U_i,measured_ is obtained in Equation (6):

(6) $${\;\;\;\;u}_{R}\left(U_{i,measured}\right)=\;\sqrt{{\left(u_{R}\left(U_{U,DMM,i}\right)\right)}^{2}+{\left(u_{R}\left(K_{VoltageProbe}\right)\right)}^{2}+{\left(u_{R}\left(\varepsilon_{DFT}\right)\right)}^{2}\;}\;$$

In addition to the sources of uncertainty listed earlier, the combined relative uncertainty of the probe includes its frequency response for frequencies up to 500 kHz—K_TF_—Type A. The contributions to the global uncertainty budget are listed in Table 4.

#### 4.1.3. Uncertainty Calculation for I_DC_

The measured DC value, I_DC,measured_, is associated with a measurement uncertainty that accounts for the combined relative uncertainties of the acquisition system (DMM)-$u_{R}(U_{I,DMM,DC})$, the combined relative uncertainties of the current probe, $u_{R}(K_{CurrentProbe})$, and the combined relative uncertainties of the resistance R_M_-$u_{R}(R_{M})$.

By applying the law of propagation of uncertainties, the combined relative uncertainty of U_DC,measured_ is obtained in (7):

(7) $${\;\;\;u}_{R}\left(I_{DC,measured}\right)=\;\sqrt{{\left(u_{R}\left(U_{I,DMM,DC}\right)\right)}^{2}+{\left(u_{R}\left(K_{CurrentProbe}\right)\right)}^{2}+\;{\left(u_{R}\left(R_{M}\right)\right)}^{2}}$$

For U_I,DMM,DC_, the standard uncertainties are the same as the ones listed in Table 3 for U_U,DMM,DC_. As for the current probe and the R_M_, they are given in Table 5.

#### 4.1.4. Uncertainty Calculation for I_i_

In addition to the standard uncertainties of I_DC,measured_, the combined uncertainties of I_i,measured_, given in Table 6, include the following: the frequency response of K for frequencies up to 500 kHz, KTF—Type A, **ε_DFT,_** and the frequency response of R_M_ up to 500 kHz which is equal to zero since we used the corrected values R_M_ with respect to frequencies.

### 4.2. On-Site Measurement System

For the field measurement chain, an identical approach has been adopted for uncertainty budget calculation with some variations in the sources of uncertainties.

A significant difference between the two chains is notable: the influence of temperature. This is a consideration of significance because the field chain is intended for use on on-site measurements and thus, is directly subjected to variations in temperature. For the estimation of temperature-related drift U_T,NI_—Type A, the field setup was placed inside an oven where the temperature was varied between 10 and 30 degrees Celsius. The voltage difference with respect to the voltage at ambient temperature was approximated.

Another important difference between the two chains is the measured frequency range. While characterizing the four voltage probes tested, we generated a square wave signal and calculated the spectrum of the acquired signal. We observed an increase in amplitude in the spectra beyond a certain frequency component around 200 kHz. Replacing the voltage probe with another type did not eliminate the phenomenon, which initially suggested that the NI card might be responsible. In a further attempt to isolate the issue, we also tested the probes with a PicoScope 4000 A and it did not exhibit this variation. However, since the Picoscope has a lower resolution (less than 24 bits) and a higher signal-to-noise ratio, it was not chosen as the setup’s acquisition system. To make sure that this phenomenon had something to do with the behavior of the NI card, a check of its datasheet did verify a greater gain between 100 kHz and 200 kHz. Further testing was conducted using sinusoidal sweeps covering frequencies between 150 and 250 kHz revealing that the gain boost begins at approximately 195 kHz. Thus, while the NI card behaves as expected up to 150 kHz, its behavior above this frequency requires further investigation and compensation. Therefore, the on-site measurement chain is tested with frequencies up to 150 kHz in alignment with the frequency intervals proposed by the IEC.

Additionally, one more parameter has an important contribution in the NI card, which is the unwanted coupling between two channels of the multi-channel board. Even though the NI card possesses a very high decoupling level, this factor is still considered during calculations: U_C,NI_—Type B.

Furthermore, for the measurement of v(t), the linearity has been calculated with input voltages up to 350 V instead of 700 V. And for the measurement of i(t), the current probe Hioki CT6710 has a conversion ratio in V/A; therefore, all the uncertainties related to the resistance R_M_ of the laboratory chain are not included in the calculations.

The contributions to the global uncertainty budget are listed in Table 7.

## 5. Synthesis of the Performances of the Two Measurement Chains

Table 8 summarizes the proposed measurement setups specifications along with their uncertainty budget results with a coverage factor of k = 2 (95.45% coverage probability):

Based on the results presented in Table 8 and their comparison with Table 1, several technical observations and conclusions can be drawn:

- **Measurement chain specifications**

The two acquisition chains proposed in this study were designed to achieve enhanced resolution and higher sampling frequencies, thereby improving acquisition accuracy and extending the studied frequency range (up to 150 kHz and 500 kHz). In this regard, both the laboratory chain (18 bits, 5 MHz) and the field chain (24 bits, 1 MHz) exhibit better specifications compared to those reported in Table 1.

- **Comparison with reference uncertainties**

At 150 kHz, the laboratory chain demonstrates uncertainty levels comparable to those of the PTB laboratory, with lower voltage and current distortions uncertainties: $7.17\times 10^{-4}$ (laboratory setup) and $2.2\times 10^{-3}$ (PTB setup) for voltage distortions uncertainties, and $1.99\times 10^{-3}$ (laboratory setup) and $4.1\times 10^{-3}$ (PTB setup) for current distortions. The voltage uncertainty of the laboratory chain could be further reduced by eliminating the voltage divider—similarly to the PTB setup—since the chosen DMM accepts direct inputs up to 1000 V.

- **Trade-off between accuracy and flexibility**

As previously noted, the laboratory setup generally achieves lower uncertainties for both DC and distortion components of voltage and current compared to the field setup. Conversely, the field setup sacrifices metrological accuracy in favor of flexibility. Its highest standard uncertainties are due to the frequency response of the voltage and current sensors. As for the NI PXIe acquisition system, it shows low uncertainties making it a flexible, accurate, and high resolution system, an appropriate fit for on-site measurements.

- **Limitations at higher frequencies**

**Figure 4:**
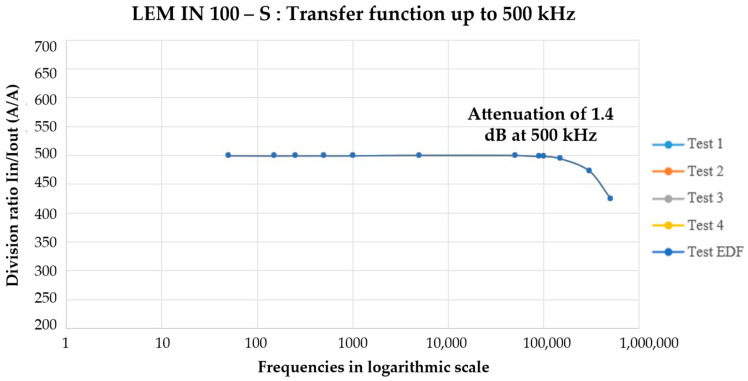
Transfer function LEM IN 100-S.

Measuring distortion components beyond 150 kHz remains a major challenge for both chains. For the laboratory configuration, the current probe can reliably operate up to 500 kHz with attenuation lower than 1.5 dB, as seen in Figure 4; nevertheless, the associated uncertainties increase by a factor of 10 compared to measurements limited to 150 kHz. Conversely,, in the field chain, electromagnetic compatibility (EMC) issues arise for frequencies > 200 kHz, which directly impacts the accurate estimation of high-frequency amplitudes. This limitation is particularly critical when assessing switching frequencies generated by electric vehicle chargers and power electronic converters greater than 200 kHz [32], where accurate spectral amplitude estimation is mandatory to ensure conformity with future electromagnetic compatibility levels.

To mitigate the high uncertainties related to the sensors frequency responses for the field setup, and the limitations of both setups performances at frequencies >150 kHz, two solutions are possible. The first is to replace the chosen sensors with devices that include built-in hardware compensation—such as Tektronix IsoVu sensors—whichmay require switching to a Tektronix acquisition system to achieve optimal performance. The second is to apply spectral error corrections, which involves refining the filtering of the NI card to suppress EMC disturbances beyond 200 kHz and compensating for the frequency response characteristics of the current and voltage sensors. This frequency response compensation is possible since repeatability tests were performed in the LNE laboratory and given in Table 4, Table 6, and Table 7 as K_Rep_, and reproducibility tests were performed in the EDF laboratory resulting in a standard deviation of the differences in measurements for the two laboratories equal to $2.63\times 10^{-5}$.

- **Impact of signal processing algorithms**

A further source of measurement uncertainty arises from the signal processing stage, particularly the windowing applied to the time-domain data. Potential solutions include the following: spectral grouping techniques, aggregating leaked energy contributions around the central frequency to obtain more accurate amplitude estimations, or alternative transforms such as the wavalet transform.

## 6. Conclusions

As highlighted in the introduction, this work aims to propose solutions to overcome DC metrological challenges. Addressing these challenges depends on two critical dimensions: the performance of the measurement chain, and the methods used for measurement and data processing. While previous initiatives have started to propose setups and methods for measuring DC distortions, our contribution focuses on solutions that offer improved resolution, reduced uncertainties, and the ability to capture higher frequency ranges. Thus, two complementary traceable measurement chains were developed, one optimized for controlled laboratory settings and the other designed to be portable for field applications. The method implemented with the measurement systems is adapted for DC distortions. Data are treated by DFT for an observation time of 200 ms with a Hamming window which leads to a better amplitude accuracy and leakage attenuation compared to other window types.

Moreover, the robustness of the on-site setup proposed has been tested across several field measurement campaigns, including the characterization of an electric vehicle’s behavior under various disturbance scenarios, as well as the evaluation of protection equipment performance for LVDC grids. A third campaign focused on assessing the impact of electrical disturbances on both AC and DC grids caused by the electric vehicle charging solution. As a result, the measurement chain has demonstrated sufficient robustness, with stable performance levels.

Overall, the measurement chains described in this paper offer effective solutions to accurately determine DC distortions in the laboratory and field and contribute to the advancement of DC metrology. Despite the challenges faced, the developed systems demonstrated comparable performances with previous proposed setups, with repeatability and reproducibility results allowing the correction of errors for uncertainties improvements. Ongoing research in measurement techniques and algorithms will help enhance the accuracy and applicability of these solutions.

## Figures and Tables

**Table 1:** Measurement systems developed for DC distortions measurement.

Components	Parameters	VSL	PTB	METAS
Voltage Divider	Ratio	201:1	NA The acquisition system can measure 1500 V	Not Specified
	Maximum Input	1000 V		
	Bandwidth	DC—150 kHz		
Current Transformer	Ratio	1500:1	1500:1	100:1 or 1000:1
	Maximum Input	1500 A	1500 A	1000 A
	Bandwidth	DC—100 kHz	DC—400 kHz	DC—150 kHz
Acquisition System	Resolution	16 bits	18 bits	16 bits
	Sampling Frequency	1 MHz	1 MHz	500 kHz
	Maximum Input	±10 V	1500 V	±10 V
Uncertainties	Voltage	For a 50 Hz AC component ${2\cdot 10}^{-4}\cdot U$	For a 150 kHz AC component ${2.2\;\cdot 10}^{-3}\cdot U$	For a 10 Hz AC component ${2.2\cdot 10}^{-4}\cdot U$
	Current	For a 50 Hz AC component ${3.6\cdot 10}^{-4}\cdot I$	For a 150 kHz AC component ${4.1\cdot 10}^{-3}\cdot I$	For a 10 Hz AC component ${6.2\;\cdot 10}^{-4}\cdot I$

**Table 3:** Standard uncertainties (k = 1) for U_DC_ in the laboratory setup.

Uncertainty Associated with	Type	Notation	Comments
Components for the DMM			
DC calibration	B	U_CE,DMM_	From the calibration certificate (CC)
Drift per year	B	U_D,DMM_	Computed based on past calibration certificates
Analog-to-digital conversion noise	A	U_Q,DMM_	From DMM characterization
Circuit noise (with cables and connections)	A	U_N,DMM_	DMM in short-circuit configuration. It is the standard deviation of the measured noise over 10,000 samples.
Influence of the temperature	B	U_T_	=0. Controlled temperature in the laboratory
Influence of the input impedance Z	B	U_Z,DMM_	Equal to the largest error from two series of measurements, corresponding to Z_DMM_ = 1 MΩ and 10 MΩ
Zero offset	B	U_offset,DMM_	=0. A zeroing function of the DMM is used
Influence of the cable lengths	B	U_cables_	Equal to the largest error from 4 series of measurements with four cable lengths: 20 cm, 50 cm, 100 cm, and 150 cm
**Components for the voltage probe**			
Calibration	B	K_CE_	From the calibration certificate (CC)
Drift per year	B	K_D_	Computed based on past calibration certificates
Linearity	A	K_L_	Standard deviation with input voltages up to 700 V
Repeatability	A	K_Rep_	Standard deviation of 5 series of repeated measurements

**Table 4:** Uncertainty budget for the measurement of u(t) with the laboratory setup.

		Uncertainty Component Related to							Combined Uncertainty	
									k = 1	k = 2
$U_{DC,measured}$	$U_{U,DMM,DC}$	$U_{CE,DMM}$	$U_{D,DMM}$	$U_{N,DMM}$	$U_{Q,DMM}$	$U_{Z,DMM}$	$U_{cables,DMM}$	$U_{offset,DMM}$	$2.81\times 10^{-4}$	$5.62\times 10^{-4}$
		$1.3\times 10^{-6}$	$1.05\times 10^{-6}$	$2.14\times 10^{-5}$	$2.31\times 10^{-6}$	$3.53\times 10^{-5}$	$2.78\times 10^{-4}$	$0.00\times 10^{-0}$		
	$K_{VoltageProbe}$	$K_{CE}$	$K_{D}$	$K_{L}$	$K_{Rep}$					
		$4.5\times 10^{-5}$	$4.33\times 10^{-7}$	$4.20\times 10^{-6}$	$1.83\times 10^{-6}$					
$U_{i,measured}$	$U_{U,DMM,i}$	$U_{CE,DMM}$	$U_{D,DMM}$	$U_{N,DMM}$	$U_{Q,DMM}$	$U_{Z,DMM}$	$U_{cables,DMM}$	$U_{offset,DMM}$	Up to 150 kHz $3.59\cdot 10^{-4}$	Up to 150 kHz $7.17\;\cdot 10^{-4}$
		$1.65\;\cdot 10^{-4}$	$1.05\times 10^{-6}$	$2.14\times 10^{-5}$	$2.31\times 10^{-6}$	$3.53\times 10^{-5}$	$2.78\cdot 10^{-4}$	$0.00\;\cdot 10^{-0}$		
	$K_{VoltageProbe}$	$K_{CE}$	$K_{D}$	$K_{L}$	$K_{Rep}$	$K_{TF}\;$(to 150 kHz)	$K_{TF}\;$(to 500 kHz)			
		$4.5\times 10^{-5}$	$4.33\times 10^{-7}$	$4.20\times 10^{-6}$	$1.83\times 10^{-6}$	$2.16\times 10^{-5}$	$1.65\;\cdot 10^{-3}$		Up to 500 kHz $3.38\;\cdot 10^{-3}$	Up to 500 kHz $6.75\;\cdot 10^{-3}$
	$\varepsilon_{DFT}$	$1.40\times 10^{-4}$								

**Table 5:** Standard uncertainties (k = 1) for IDC in the laboratory setup.

Associated with	Type	Notation	Comments
Components for the current probe			
Calibration	B	K_CE_	From the calibration certificate (CC)
Drift per year	B	K_D_	=0. Since the LEM was bought within the last 6 months
Linearity	A	K_L_	Standard deviation of the linearity with input currents up to 30 A
Repeatability	A	K_Rep_	Standard deviation of 5 series of repeated measurements
Influence of the conductor position	B	K_Position_	Standard deviation with respect to conductor position inside the LEM sensor
**Components for the resistance R_M_**			
Calibration	B	R_M,CE_	From the calibration certificate (CC)

**Table 6:** Uncertainty budget of the measurement of i(t) with the laboratory setup.

		Uncertainty Component Related to							Combined Uncertainty	
									k = 1	k = 2
$I_{DC,measured}$	$U_{I,DMM,DC}$	$U_{CE,DMM}$	$U_{D,DMM}$	$U_{N,DMM}$	$U_{Q,DMM}$	$U_{Z,DMM}$	$U_{cables,DMM}$	$U_{offset,DMM}$	$4.15\times 10^{-4}$	$8.31\times 10^{-4}$
		$1.3\times 10^{-6}$	$1.05\times 10^{-6}$	$2.14\times 10^{-5}$	$2.31\times 10^{-6}$	$3.53\times 10^{-5}$	$2.78\times 10^{-4}$	$0.00\times 10^{-0}$		
	$K_{CurrentProbe}$	$K_{CE}$	$K_{D}$	$K_{L}$	$K_{Rep}$	$K_{Position}$				
		$5.00\times 10^{-6}$	$0.00\times 10^{-0}$	$2.77\times 10^{-4}$	$1.10\times 10^{-4}$	$6.92\times 10^{-5}$				
	$R_{M}$	$R_{M,CE}$	$R_{M,T}$							
		$1.00\times 10^{-6}$	$0.00\times 10^{-0}$							
$I_{i,measured}$	$U_{I,DMM,i}$	$U_{CE,DMM}$	$U_{D,DMM}$	$U_{N,DMM}$	$U_{Q,DMM}$	$U_{Z,DMM}$	$U_{cables,DMM}$	$U_{offset,DMM}$	Up to 150 kHz $9.96\;\times 10^{-4}$	Up to 150 kHz $1.99\;\times 10^{-3}$
		$1.65\times 10^{-4}$	$1.05\times 10^{-6}$	$2.14\times 10^{-5}$	$2.31\times 10^{-6}$	$3.53\times 10^{-5}$	$2.78\times 10^{-4}$	$0.00\times 10^{-0}$		
	$K_{CurrentProbe}$	$K_{CE}$	$K_{D}$	$K_{L}$	$K_{Rep}$	$K_{Position}$	*K_TF_* (to 150 kHz)	*K_TF_* (to 500 kHz)		
		$5.00\times 10^{-6}$	$0.00\times 10^{-0}$	$2.77\times 10^{-4}$	$1.10\times 10^{-4}$	$6.92\times 10^{-5}$	$8.79\times 10^{-4}$	$3.92\times 10^{-2}$	Up to 500 kHz $3.92\;\times 10^{-2}$	Up to 500 kHz $7.84\;\times 10^{-2}$
	$R_{M}$	$R_{M,CE}$	$R_{M,T}$	$R_{M,f}$						
		$1.00\times 10^{-6}$	$0.00\times 10^{-0}$	$0.00\times 10^{-0}$						
	$\varepsilon_{DFT}$	$1.40\times 10^{-4}$								

**Table 7:** Uncertainty budget for the measurements of u(t) and i(t) with the on-site setup.

		Uncertainty Component Related to					Combined Uncertainty	
							k = 1	k = 2
$U_{DC,measured}$	$U_{U,NI,DC}$	$U_{N,NI}$	$U_{Q,NI}$	$U_{C,NI}$	$U_{offset,NI}$	$U_{T}$	$1.87\times 10^{-3}$	$3.73\times 10^{-3}$
		$1.25\times 10^{-5}$	$1.71\times 10^{-8}$	$1.00\times 10^{-5}$	$1.44\times 10^{-4}$	$9.9\times 10^{-5}$		
	$K_{VoltageProbe}$	$K_{L}$	$K_{Rep}$					
		$1.86\times 10^{-3}$	$7.43\times 10^{-5}$					
$U_{i,measured}$	$U_{U,NI,i}$	$U_{N,NI}$	$U_{Q,NI}$	$U_{C,NI}$	$U_{offset,NI}$	$U_{T}$	$8.36\times 10^{-3}$	$1.67\times 10^{-2}$
		$1.25\times 10^{-5}$	$1.71\times 10^{-8}$	$1.00\times 10^{-5}$	$1.44\times 10^{-4}$	$9.9\times 10^{-5}$		
	$K_{VoltageProbe}$	$K_{L}$	$K_{Rep}$	$K_{TF}$				
		$1.86\times 10^{-3}$	$7.43\times 10^{-5}$	$8.15\times 10^{-3}$				
	$\varepsilon_{DFT}$	$1.40\times 10^{-4}$						
$I_{DC,measured}$	$U_{I,NI,DC}$	$U_{N,NI}$	$U_{Q,NI}$	$U_{C,NI}$	$U_{offset,NI}$	$U_{T}$	$2.06\times 10^{-3}$	$4.12\times 10^{-3}$
		$1.25\times 10^{-5}$	$1.71\times 10^{-8}$	$1.00\times 10^{-5}$	$1.44\times 10^{-4}$	$1.15\times 10^{-3}$		
	$K_{CurrentProbe}$	$K_{L}$	$K_{Rep}$					
		$1.48\times 10^{-3}$	$8.36\times 10^{-4}$					
$I_{i,measured}$	$U_{I,NI,i}$	$U_{N,NI}$	$U_{Q,NI}$	$U_{C,NI}$	$U_{offset,NI}$	$U_{T}$	$6.83\times 10^{-3}$	$1.37\times 10^{-2}$
		$1.25\times 10^{-5}$	$1.71\times 10^{-8}$	$1.00\times 10^{-5}$	$1.44\times 10^{-4}$	$1.15\times 10^{-3}$		
	$K_{CurrentProbe}$	$K_{L}$	$K_{Rep}$	$K_{TF}$				
		$1.48\times 10^{-3}$	$8.36\times 10^{-4}$	$6.52\times 10^{-3}$				
	$\varepsilon_{DFT}$	$1.40\times 10^{-4}$						

**Table 8:** Performances of the two measurement chains.

Component	Parameters	Laboratory Setup	On-Site Setup
Voltage Sensor	Ratio	1000:0.8	100:1
	Maximum input	1000 V	400 V
	Bandwidth	DC—1 MHz	DC—30 MHz
Current Sensor	Ratio	500:1	0.1 (V/A)
	Maximum input	100 A	30 A
	Bandwidth	DC—2 MHz	DC—50 MHz
Acquisition System	Resolution	18 bits	24 bits
	Sampling frequency	5 MHz	1 MHz
	Maximum input	1000 V	±10 V
Confidence Interval	U_DC_	$U_{DC,measured}\pm 5.62\times 10^{-4}\cdot U_{DC,measured}$	$U_{DC,measured}\pm {3.73\times 10}^{-3}\cdot U_{DC,measured}$
	U_i_	$U_{i,measured}\pm 7.17\times 10^{-4}\cdot U_{i,measured}$ (to 150 kHz)	$U_{i,measured}\pm {1.67\times 10}^{-2}\cdot U_{i,measured}$ (to 150 kHz)
		$U_{i,measured}\pm 6.75\times 10^{-3}\cdot U_{i,measured}$ (to 500 kHz)	
	I_DC_	$I_{DC,measured}\pm 8.31\times 10^{-4}\cdot I_{DC,measured}$	$I_{DC,measured}\pm 4.12\times 10^{-3}\cdot I_{DC,measured}$
	I_i_	$I_{i,measured}\pm 1.99\times 10^{-3}\cdot I_{i,measured}$ (to 150 kHz)	$I_{i,measured}\pm 1.37\times 10^{-2}\cdot I_{i,measured}$ (to 150 kHz)
		$I_{i,measured}\pm 7.84\times 10^{-2}\cdot I_{i,measured}$ (to 500 kHz)	

## Data Availability

The raw data supporting the conclusions of this article will be made available by the authors on request.
